# The atherogenic and metabolic impact of non-HDL cholesterol versus other lipid sub-components among non-diabetic and diabetic Saudis

**DOI:** 10.1186/1476-511X-6-9

**Published:** 2007-04-04

**Authors:** Nasser M Al-Daghri, Omar S Al-Attas, Khalid Al-Rubeaan

**Affiliations:** 1 College of Science Biochemistry Department, King Saud University, Riyadh, Kingdom of Saudi Arabia; 2 King Saud University, College of Medicine, Medicine Department, Saudi Arabia Riyadh, Kingdom of Saudi Arabia

## Abstract

**Background:**

Several trials from different populations have reported that non-high density lipoprotein cholesterol (non-HDL-C) has more predictive power than low-density lipoprotein cholesterol (LDL-C) in detecting coronary heart disease (CHD) and none in any Arab community whose propensity to develop CHD is higher compared to other ethnicities. This study aims to determine and compare the impact of non-HDL-C versus other lipid parameters, in predicting coronary heart disease among diabetic versus non-diabetic adult Saudis and identify the lipid parameters which make a significant contribution in the development of coronary heart disease, diabetes mellitus, and metabolic syndrome. 733 adult Saudis were recruited and divided into groups of diabetics and non-diabetics. Each participant completed a questionnaire, underwent physical exam including 12-L ECG, and submitted a fasting blood sample where glucose and lipid parameters were analyzed using routine procedures.

**Results:**

462 subjects (age 45.03 ± 11.52; BMI 28.91 ± 6.07) were classified non-diabetics while the remaining 271 (age 52.73 ± 11.45, BMI 30.15 ± 6.62) were diabetics. 99 out of 465 (21.3%) of non-diabetics had CHD and 114 out of 271 (52.5%) in the diabetics. Non-HDL cholesterol was the best predictor among the non-diabetics (odds-ratio 2.89, CI 1.10–7.58, p-0.03). Total cholesterol was the highest single predictor for the development of CHD among the lipids (odds-ratio 1.36, CI 0.68–2.71, p-0.39) but HDL-cholesterol although small was significant (odds-ratio 0.52, CI 0.27–0.99, p-0.05).

**Conclusion:**

This study supports the use of non-HDL cholesterol as the more practical and reliable target for lipid lowering therapy among the Saudi population.

## Background

It is apparent that atherosclerosis manifested by coronary heart disease (CHD) is not only the single most common cause of death among middle-aged people in industrialized nations, but it is also the leading cause of morbidity and mortality among people suffering from diabetes mellitus. Coronary heart disease and diabetes mellitus (DM) are both chronic metabolic diseases whose pathophysiology remains extremely complex and multi-factorial. Yet of the many risk factors to blame, much attention has focused on the elevated lipid profile and its "atherogenic potential" as a very powerful risk factor for the aggravation of these diseases. In the recent report of the expert panel from the National Cholesterol Education Program (NCEP) on detection, evaluation and treatment of high blood cholesterol in adults (Adult Treatment Panel III), they continued to recognize low-density lipoprotein cholesterol (LDL-C) as the primary target for cholesterol lowering therapy [1]. This was of course based on the premise that keeping lipid parameters to their optimal levels would be certain to lessen the chances of developing coronary heart disease.

Emerging novel risk factors were nevertheless recognized by NCEP. One of them is the non-high density lipoprotein cholesterol (non-HDL-C), whose value can be calculated by subtracting HDL-cholesterol from the total cholesterol. While several trials from different populations have reported that non-HDL-C has more predictive power than LDL-C in detecting CHD [2,3], no such trials have so far been made in any Arab community whose propensity to develop coronary heart disease is high compared to other ethnicities [4]. Hence, this study primarily aims to determine and compare the power and influence of the novel risk factor non-HDL- cholesterol versus other lipid parameters, in predicting coronary heart disease among diabetic versus non-diabetic adult Saudis. This study will also attempt to identify which among the lipid parameters makes a significant contribution in the development of coronary heart disease, diabetes mellitus, and metabolic syndrome in the same population.

## Results

From the sample population, 462 (age 45.03 ± 11.52; BMI 28.91 ± 6.07) were classified as non-diabetic, while the remaining 271 (age 52.73 ± 11.45, BMI 30.15 ± 6.62) were categorized as diabetic.

Table 1 reveals the clinical and metabolic characteristics of both groups. It is apparent that the diabetics were significantly older and fatter, with a higher blood pressure and elevated lipid profile, except for HDL-cholesterol, than the non-diabetic group. Only LDL-cholesterol was non-significant.

**Table 1:** Clinical and Metabolic characteristics of subjects

**Variable**	**Non-Diabetics**	**Diabetics**	**P-Value**
N	462	271	
Age (years)	45.03 ± 11.52	52.73 ± 11.45	**0.000**
Systolic BP (mmHg)	120.57 ± 18.23	130.17 ± 19.74	**0.000**
Diastolic BP (mmHg)	78.15 ± 11.65	82.27 ± 10.71	**0.000**
BMI (kg/m^2^)	28.91 ± 6.07	30.15 ± 6.62	**0.011**
LDL (mmol/L)	3.63 ± 1.31	3.47 ± 1.55	0.17
HDL (mmol/L)	0.91 ± 0.45	0.80 ± 0.32	**0.000**
Non-HDL (mmol/L)	4.36 ± 1.47	4.67 ± 1.54	**0.009**
Total Cholesterol (mmol/Ll)	5.26 ± 1.39	5.52 ± 1.74	**0.04**
Triglycerides (mmol/L)	1.74 ± 1.06	2.63 ± 1.37	**0.000**

* Data are presented as mean ± SD; p-value significant at < 0.05

In stepwise regression, triglycerides (R^2 ^0.12, p-0.000) and HDL-cholesterol (R^2 ^0.13 p-0.003) had the significant contribution when diabetes mellitus was regarded as the dependent variable. Triglyceride was also the dominant lipid (R^2 ^0.007, p-0.03) in coronary heart disease. At the same time, metabolic syndrome as defined by NCEP ATPIII had triglycerides, HDL-cholesterol and total cholesterol as its significant contributors (R^2 ^0.17, 0.22, 0.23, p-value 0.000 respectively).

Table 2 shows that 99 out of 465 (21.3%) of non-diabetics had CHD as opposed to 114 out of 271 (52.5%) among the diabetics indicating diabetes as a very strong independent risk factor for the development of CHD among Saudis.

**Table 2:** Relative risk of probable coronary heart disease for various cut-off values of lipids

	**Non-Diabetic**	**Diabetic**
N	465	271
Probable CHD	99	114
**LDL cholesterol (mmol/L)**		
< 2.59	0.87 (0.70–1.09)	0.95 (0.86–1.05)
2.59–3.34	0.99 (0.76–1.30)	1.03 (0.91–1.17)
≥ 3.35	1.05 (0.92–1.19)	1.13 (0.91–1.39)
**Non-HDL- cholesterol (mmol/L)**		
< 3.35	0.94 (0.85–1.04)	0.95 (0.74–1.22)
3.35–4.13	0.86 (0.67–1.09)	1.06 (0.93–1.21)
≥ 4.14	1.01 (0.92–1.11)	1.15 (0.93–1.42)
**Total Cholesterol (mmol/L)**		
< 5.17	1.02 (0.93–1.12	0.81 (0.65–1.00)
5.17–6.19	0.93 (0.84–1.03)	1.11 (0.86–1.44)
≥ 6.20	1.09 (0.95–1.24)	1.18 (0.91–1.53)
**HDL Cholesterol (mg/dl)**		
≥ 1.55	0.85 (0.76–0.96)	1.76 (0.56–5.47)
1.03–1.54	1.10 (0.96–1.25)	1.37 (0.98–1.91)
< 1.03	0.99 (0.89–1.10)	0.70 (0.51–0.97)
**Triglycerides (mg/dl)**		
< 1.69	0.79 (0.70–0.89)	0.96 (0.75–1.23)
1.69–2.25	0.98 (0.86–1.11)	1.20 (0.86–1.67)
≥ 2.26	1.05 (0.93–1.19)	0.94 (0.75–1.46)

*Data presented as Relative risk (95% Confidence interval)

In general, the chances of developing coronary heart disease increase as individual lipid components increase in both groups. Worth noting is the positive trend for HDL cholesterol in which the relative risk of CHD was highest at a ≥ 1.55 level among the diabetics [1.76 (0.56–5.47)].

In logistic regression analysis, the cut-off points used for each lipid parameter were based on the values set by NCEP ATP III, which they considered optimal. Thus, the cut-off points were: ≥ 2.59 mmol/L (≥ 100 mg/dl) in LDL-C, ≥ 3.35 mmol/L (≥ 130 mg/dl) for non-HDL-C, ≥ 1.69 mmol/L (≥ 150 mg/dl) in triglycerides and ≤ 1.03 mmol/L (≤ 40 mg/dl) in HDL-C and ≥ 5.17 mmol/L (≥ 200 mg/dl) in total cholesterol. Clearly the non-HDL cholesterol was the best predictor among the non-diabetics (odds-ratio 2.89, CI 1.10–7.58, p-0.03) (see Table 3). In the diabetics group, total cholesterol was the highest single predictor for the development of CHD among the lipids (odds-ratio 1.36, CI 0.68–2.71, p-0.39) but HDL-cholesterol has a small but significant effect (odds-ratio 0.52, CI 0.27–0.99, p-0.05) (see Table 4).

**Table 3:** Logistic regression analysis using probable CHD as a dependent variable and the various lipids as independent variables

**Non-Diabetics**					
**Predictor**	**β**	**SE**	**Odds-Ratio**	**95% Confidence Interval**	**P-Value**

Non-HDL	1.06	0.49	2.89	1.10–7.58	0.03
HDL	-0.19	0.27	0.83	0.49–1.40	0.48
LDL	-0.65	0.48	0.52	0.21–1.32	0.17
Triglycerides	0.07	0.25	1.07	0.65–1.76	0.79
Total Cholesterol	-0.36	0.29	0.69	0.39–1.23	0.21

β – Beta; SE – standard error

**Table 4:** Logistic regression analysis using probable CHD as a dependent variable and the various lipids as independent variables

**Diabetics**					
**Predictor**	**β**	**SE**	**Odds-Ratio**	**95% Confidence Interval**	**P-Value**

Non-HDL	-0.26	0.51	0.77	0.28–2.01	0.61
HDL	-0.66	0.33	0.52	0.27–0.99	0.05
LDL	0.16	0.47	1.18	0.47–2.93	0.73
Triglycerides	0.09	0.31	1.09	0.59–2.01	0.78
Total Cholesterol	0.31	0.35	1.36	0.68–2.71	0.39

β – Beta; SE – standard error

## Discussion

Grundy, in a recent report, emphasizes that there has to be strong evidence of superiority for non-HDL cholesterol to be regarded as the primary target of lipid therapy [6]. Since then, clinical trials all over the world have made further contributions in the challenge to discover whether non-HDL cholesterol is indeed superior to the incumbent LDL cholesterol as the primary target of lipid-lowering therapy [7-10]. This study can be regarded as contributing to this growing evidence, using Saudis as the first subjects to be considered in the Arab population as a whole.

The superiority of non-HDL cholesterol over LDL-cholesterol started from a comparison of equations; non-HDL cholesterol is a simple subtraction while LDL-cholesterol is derived from 3 different analytes, with a conversion factor assumption. From a practical point of view, it is easier and faster to compute for non-HDL cholesterol not to mention the unreliability of LDL-cholesterol values when ≥ 400 mg/dl of serum triglycerides is reached [11]. Arguably, LDL cholesterol remains to be the major player in atherogenesis, but, when inaccuracy sets in, the validity of clinical management will also be open to question. Several studies support the view that non-HDL cholesterol and apo B are superior to LDL cholesterol, especially among diabetics whose triglyceride levels exceed the accuracy limit of the Friedewald formula for LDL-cholesterol [11,12].

A study by Liu and his colleagues concludes that non-HDL cholesterol is a stronger predictor of CHD death among those with diabetes than among those with LDL cholesterol and should be given more consideration in the clinical approach to risk reduction among diabetic patients [8]. Contrary to their findings, our study suggests non-HDL as a stronger predictor among non-diabetics rather than diabetics, in terms of developing CHD. The difference in results can be explained by several factors. First among these factors is the choice of dependent variable and the number of samples used; their study utilized CHD death as the dependent variable with 19,381 samples while our study focused more on existing CHD as the dependent variable with 733 samples. The great discrepancy in sample size and the unequal distribution of non-diabetics (462) to diabetics (271) in the present study may also have contributed to the disagreement between the findings. Nevertheless, the potential significance of non-HDL as a clinical tool in the management of non-diabetic patients merits supplementary investigation. Furthermore, in their report there is a negative association of HDL with CHD, which is different from our results. This could be explained by the fact that Arabs, Saudis in particular, have a lower prevalence of hypercholesterolemia than do their American and European counterparts [13]. In addition, we also considered the younger population used, together with the culture and lifestyle differences, one of which is the total prohibition of alcoholic beverages in the Kingdom, which can greatly alter the lipid and coagulation profiles of the subjects used [14,15]. As evidenced by the results of this study, decreased HDL-cholesterol levels may not be as powerful as the rest of their lipid counterparts when it comes to predicting CHD, but its contribution to the progression of diabetes mellitus and metabolic syndrome in the Saudi population is nevertheless equally important.

In our study, elevated triglycerides were the consistent single significant contributor to the development of CHD, diabetes mellitus and metabolic syndrome among the rest of the lipid sub-components. While it is apparent that hypertriglyceridemia is more closely linked to the constellation of abnormalities which constitutes metabolic syndrome, the exact atherogenic properties of triglycerides have been hard to explain, perhaps secondary to the greater biologic variance than cholesterol [16]. Williams and his colleagues report that elevated triglycerides is a common abnormality in patients who had myocardial infarction [17], while Benfante et.al confirm that triglyceride value in those below 60 years was an independent predictor of CHD, but not in older people [18]. The mean age of our subjects fall within the cut-off set by the latter's study, which probably explains why triglycerides played a significant part in the pathogenesis of these chronic diseases. The association of triglycerides with coronary heart disease remains difficult to unravel. Elevated levels do not necessarily indicate increased atherogenicity suggesting that only certain components may be atherogenic or may be associated with metabolic abnormalities which are atherogenic [16].

This study measured the lipid profiles of non-diabetic and diabetic Saudi subjects to assess the impact of individual lipid parameters, as compared to non-HDL cholesterol, in predicting coronary heart disease. Given the fact that non-HDL cholesterol possesses all the atherogenic lipoproteins (VLDL, intermediate-density lipoprotein and LDL) as opposed to LDL alone [8], the possibility that it is superior to LDL in CHD risk prediction is undoubtedly strong. Coronary heart disease is the end product of a chronic interplay of metabolic and environmental influences requiring the element of time, which is not modifiable. Early detection and intervention, therefore, using clinically important parameters such as non-HDL cholesterol are vital to overall success in the management of CHD.

This study acknowledges some limitations. The significant age gap in this study aside from the co-existing morbidity which is diabetes, has undoubtedly contributed much of the difference in relative risks of both the non-diabetic and diabetic subjects. Other confounding factors in the development of CHD which were not controlled in this study, such as smoking, the presence of hypertension, obesity, gender and family history warrant additional investigation. Nevertheless, this study acknowledges the fact that lipids play an essential role in atherogenesis, and that it is accelerated in patients with diabetes mellitus. It is the authors' hope that this study will be of help in the future assessment of international authorities such as NCEP in acknowledging novel risk factors such as non-HDL cholesterol as a potent risk factor which should be emphasized in the prevention of coronary heart disease through lipid lowering agents.

## Conclusion

In summary, elevated non-HDL-cholesterol has the highest risk of CHD among non-diabetics while elevated total cholesterol has the highest risk among the diabetics. Hypertriglyceridemia is the common significant contributor for the development of CHD, DM and metabolic syndrome. This study supports the use of non-HDL cholesterol as the more practical and reliable target of lipid lowering therapy among the Saudi population.

## Methods

A total of 733 adult Saudis who were attending the out-patient department of the Diabetes Research Unit of King Abdul-Aziz University Hospital, Riyadh, Kingdom of Saudi Arabia were recruited in this prospective and cross-sectional study, which was conducted in 2005. Each participant was given a generalized questionnaire which included personal information and past and present medical history. They were subdivided into two groups: non-diabetics and diabetics. Diagnosis of diabetes was established if the patient had: 1) prior diagnosis of type 2 diabetes mellitus; 2) fasting plasma glucose (FPG) was > 7.0 mmol/L and/or if taking oral hypoglycemics or on insulin therapy; 3) clinical manifestations of diabetes (polydipsia, polyphagia, polyuria) and/or 4 2-hour oral glucose tolerance test (OGTT) ≥ 11.1 mmol/L for asymptomatic patients with elevated FPG. Patients with unstable conditions, such as poorly controlled diabetes with complications, were excluded. All participants underwent complete physical examination, including blood pressure, height, weight, waist and hip measurements. BMI was calculated as weight in kilograms divided by height in squared meters. They also submitted fasting blood samples the lipid profiles of which were measured, including fasting plasma glucose under routine laboratory procedures. LDL-cholesterol was calculated using the Friedewald formula [11] while non-HDL cholesterol was calculated by the difference between total and HDL-cholesterol. 12-L ECG was also given and CHD was diagnosed on the basis of prior diagnosis, abnormal resting ECG (pathological Q-waves, T-wave inversion, etc...) and/or a history of coronary angiography [5]. Written consent and approval were obtained prior to being included in the study. Ethical approval was granted by the Ethics Committee of the College of Medicine and Research center of King Saud University, Riyadh, Kingdom of Saudi Arabia, prior to the research proper.

### Metabolic syndrome

Each subject was screened for the presence of metabolic syndrome based on the criteria set by NCEP ATPIII, which required at least 3 risk factors for establishing diagnosis. The following risk factors were considered: fasting plasma glucose ≥ 110 mg/dl; blood pressure ≥ 130/85 mmHg or on anti-hypertensive treatment; plasma triglycerides ≥ 1.7 mmol/L; plasma HDL cholesterol < 1.04 mmol/L in men and < 1.3 mmol/L in women; and waist circumference ≥ 102 cm in men and ≥ 88 cm in women. A total of 385 subjects (52.5%) had metabolic syndrome; 183 of whom were non-diabetics and 202 diabetics (not shown in table).

### Statistical analysis

SPSS version 11.5 (Chicago, Illinois) for Windows was used for the statistical evaluation of the results obtained. All data are presented as mean ± SD since all variables of interest were normally distributed. An independent student's t-test was used to compare the variables from both groups. Relative risk was utilized to assess the probability of acquiring possible coronary heart disease at different levels of each lipid profile component. Logistic regression analysis was used to assess risk for individual components of the lipid profile among the diabetics and non-diabetics. Stepwise linear regression was used to determine the contribution of individual lipid parameters in the development of diabetes, coronary heart disease and metabolic syndrome.

## Competing interests

The author(s) declare that they have no competing interests.

## Authors' contributions

NA for the concept and design; OA for the drafting and revising of the manuscript; KA for the acquisition and interpretation of data.
